# Regulation of gene expression in chickens by heat stress

**DOI:** 10.1186/s40104-020-00523-5

**Published:** 2021-01-11

**Authors:** Akshat Goel, Chris Major Ncho, Yang-Ho Choi

**Affiliations:** 1 Department of Animal Science, Gyeongsang National University, Jinju, 52828 Republic of Korea; 2 Division of Applied Life Sciences (BK21 Plus Program), Gyeongsang National University, Jinju, 52828 Republic of Korea; 3 Institute of Agriculture and Life Sciences, Gyeongsang National University, Jinju, 52828 Republic of Korea

**Keywords:** Antioxidant, Gene expression, Heat stress, Immunity, Metabolism, Nutrient transporter, Poultry

## Abstract

**Abstract:**

High ambient temperatures are a critical challenge in the poultry industry which is a key producer of the animal-based food. To evaluate heat stress levels, various parameters have been used, including growth rates, blood metabolites, and hormones. The most recent advances have explored expression profiling of genes that may play vital roles under stress. A high ambient temperature adversely affects nutrient uptake and is known to modulate the expression of genes encoding for sodium-dependent glucose transporters, glucose transporters, excitatory amino acid transporters, and fatty acid-binding proteins which are responsible for the absorption of macronutrients in the intestine. Various defensive activities are stimulated to protect the cell of different tissues from the heat-generated stress, including expression of early stress response genes coding for heat shock protein (HSP), c-FOS like protein, brain-derived neurotrophic factor (BDNF), and neuronal nitric oxide synthase (nNOS); antioxidant enzyme genes such as superoxide dismutase (SOD), catalase (CAT), and nicotinamide adenine dinucleotide phosphate oxidase (NOX4); and immune-related genes such as cytokines and toll-like receptors (TLRs). The potential role of HSPs in protecting the cell from stress and their presence in several tissues make them suitable markers to be evaluated under heat stress. BDNF and c-FOS genes expressed in the hypothalamus help cells to adapt to an adverse environment. Heat causes damage to the cell by generating reactive oxygen species (ROS). The NOX4 gene is the inducer of ROS under heat stress, which is in turns controlled by antioxidant enzymes such as SOD and CAT. TLRs are responsible for protecting against pathogenic attacks arising from enhanced membrane permeability, and cytokines help in controlling the pathogen and maintaining homeostasis. Thus, the evaluation of nutrient transporters and defense mechanisms using the latest molecular biology tools has made it possible to shed light on the complex cellular mechanism of heat-stressed chickens. As the impacts of heat stress on the above-mentioned aspects are beyond the extent to which the reduced growth performance could be explained, heat stress has more specific effects on the regulation of these genes than previously thought.

**Graphical abstract:**

Effect of heat exposure on the nutrient transporters, antioxidants, and immune inflammation in chickens. Most of the nutrient transporters were suppressed under heat stress. Increase in the production of reactive oxygen species resulted in enhanced production of antioxidant enzymes. Expression of various proinflammatory cytokines and toll-like receptors were enhanced due to heat stress in chicken. 
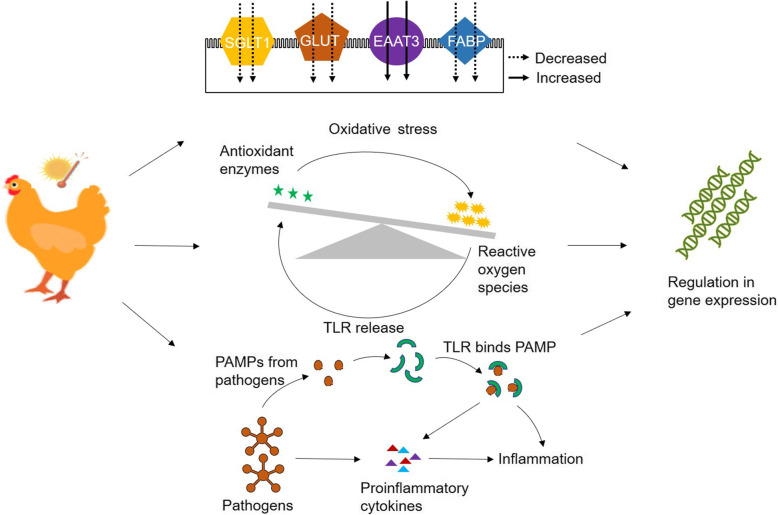

## Introduction

**Fig. 1:**
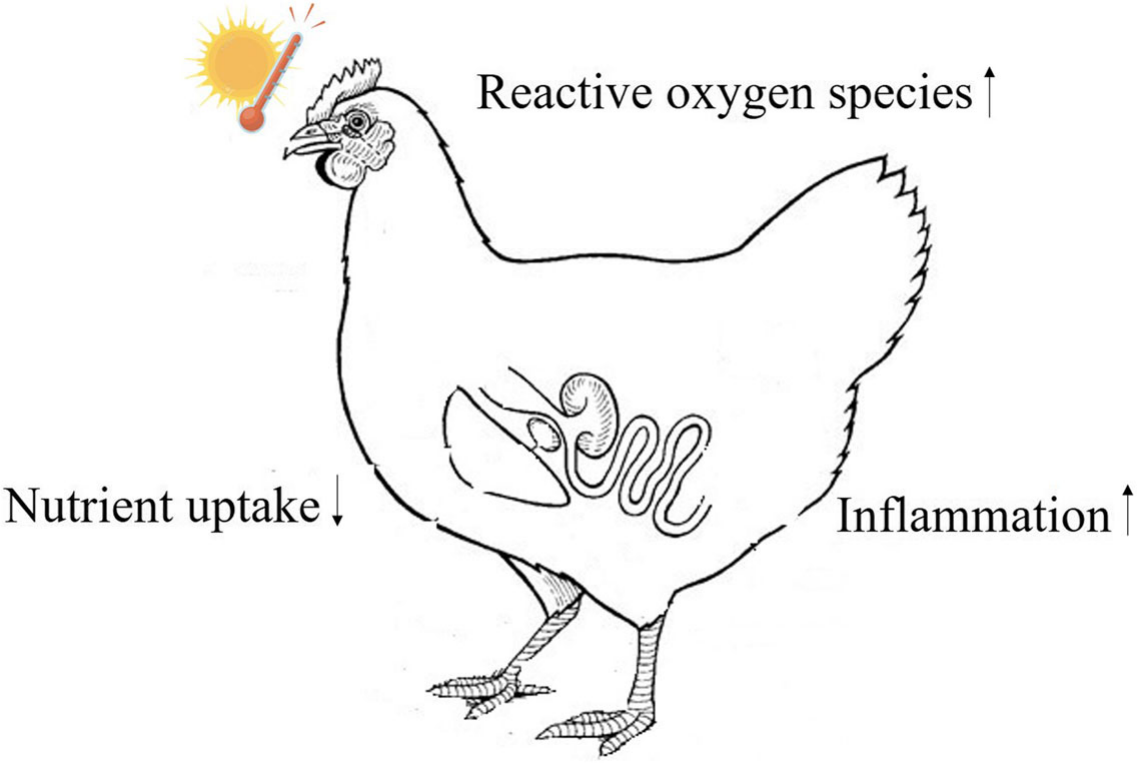
Effect of heat stress on the chicken. Enhancement in the production of reactive oxygen species and immune inflammation and reduction in the nutrient uptake occurs in the chickens reared under high ambient temperature. The diagram is drawn based on the articles [1–4]

Animals are sensitive organisms that face environmental stress due to global warming. High environmental temperatures have severe effects on production performance in livestock animals raised for animal protein (Fig. 1). The poultry industry is one of the largest among the industries that have been adversely affected by increased environmental temperature. High ambient temperature beyond the thermoneutral range is one of the most lethal stressors for raising poultry in the present scenario [1]. Deterioration of chicken growth rates, feed intake, and meat yield are the most common complications associated with high environmental exposure [2]. Modulation of blood metabolites and hormone levels is usually observed when birds are raised under high ambient temperature. Previously, most studies have used these parameters as markers to evaluate the effect of heat stress. Recent advances in the field of biotechnology have made it possible to explore the effects of heat stress “on molecular levels” [5–7]. Evaluation of gene expression provides insights into how the cells respond to heat stress. Although protein expression is equally important, contradictions between the expression of gene and protein levels have been reported previously [8, 9].

High ambient temperature results in decreased feed intake and compromised intestinal health [8, 10]. To assess the impact of heat stress on gut health, exploration of nutrient transporter genes can be an effective way to understand the role of nutrient uptake [9, 11]. Immune suppression is another disadvantage of temperature modulation. Alterations in the levels of various immune marker genes, including interleukins (ILs), TLRs, and tumor necrosis factors (TNFs) have been reported in the spleen and intestine of chicks when exposed to high ambient temperature [6, 12]. Oxidative status is the principal issue that emerges due to heat stress [3]. Further, the direct correlation of muscle with growth performance increases its importance in poultry production. The excessive production of reactive oxygen species (ROS) causes oxidative injury in the mitochondria of avian muscle cells, resulting in decreased body weight gain in heat-stressed chickens [2, 4]. The origin of oxidative injury could be mediated through the upregulation of NADPH oxidase 4 (NOX4) gene and downregulation of the avian uncoupling protein (avUCP) gene in muscle cells [13, 14]. Superoxide dismutase (SOD), catalase (CAT), and glutathione peroxidase (GPX) are the major antioxidant systems that encounter oxidative stress. The excessive production of ROS is controlled by different antioxidant enzymes in a series of steps. SOD breaks down free radicals to hydrogen peroxide, while CAT and GPX break down hydrogen peroxide to water and molecular oxygen [15, 16]. The use of gene expression profiles enables higher accuracy for strategic evaluation of modulations under temperature variations.

The poultry industry is facing threats due to a steady increase in environmental temperature. Advances in biotechnology have opened the way to explore the alterations in genes arising from continuous exposure of birds to heat stress. A strong association between reduced feed intake and retarded growth rate with heat stress encouraged us to review nutrient transporters. Further, defense mechanisms such as immune functions are also affected by heat stress. We have tried to discuss the initial hypothalamic responses followed by antioxidant and immune response in these regards. To the best of our knowledge, no review has been conducted to evaluate the present knowledge on the expression profiles of genes related to nutrient uptake, immunity, antioxidant and metabolic status after heat stress. This review summarizes the role of these genes and their expression modulation under temperature inflection. Further, it provides novel insights to improve our understanding regarding the effects of temperature enhancement on the core fundamentals of gut physiology and immune enhancement. Here, updates on the role of genes and their expression profile under heat stress in domesticated birds have been documented.

## Nutrient transporters under heat stress

**Fig. 2:**
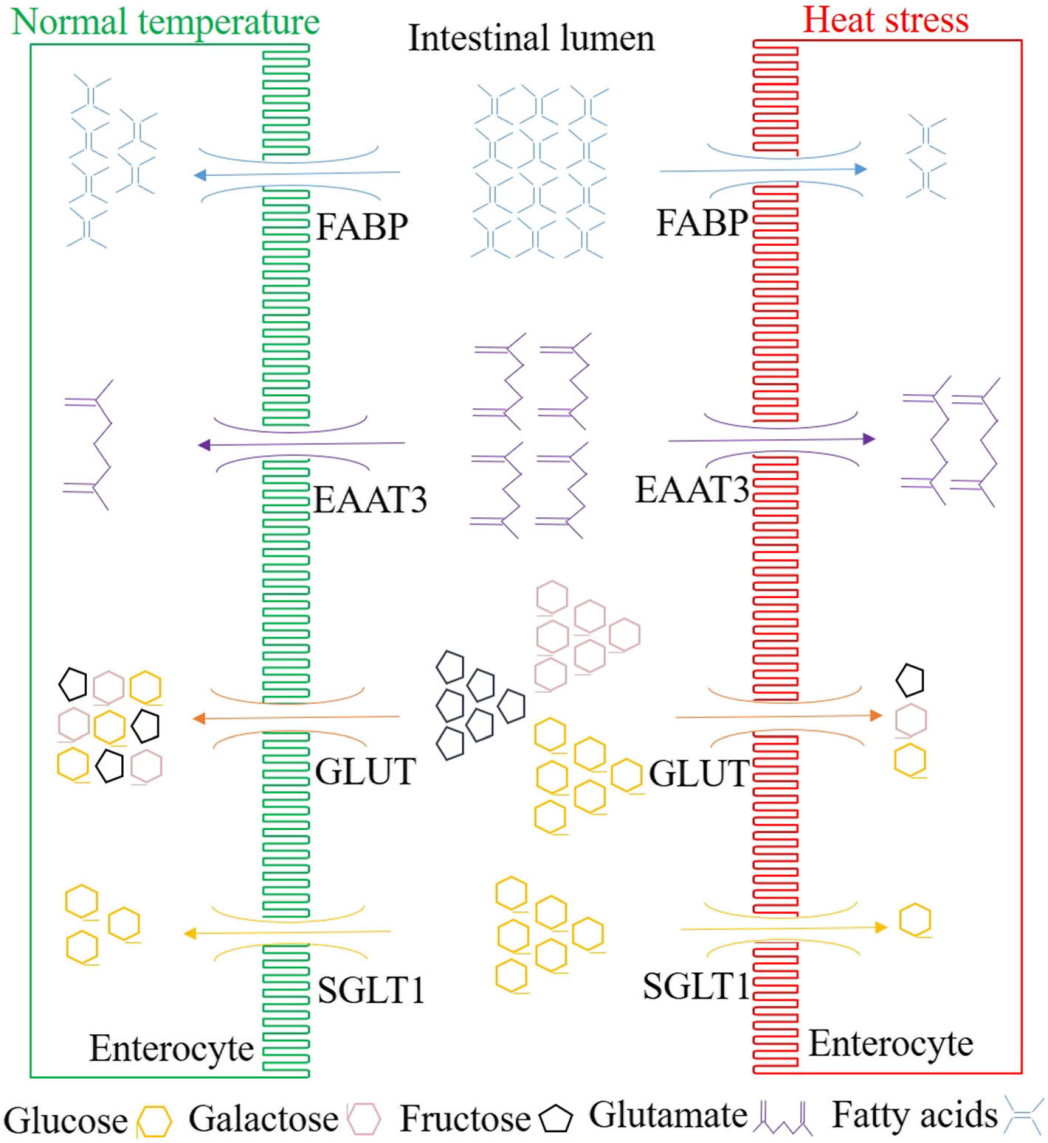
Modulation of nutrient transporters in the chicken gut under heat stress. The expression of nutrient transporter genes such as SGLT, GLUT, and FABP is decreased while the expression of the EAAT gene is increased when chickens are exposed to heat stress. The uptake of nutrients is affected by their respective transporters under heat stress. The diagram is drawn based on the articles [5, 9]. SGLT: Sodium-dependent glucose transporter; GLUT: Glucose transporter; EAAT: Excitatory amino acid transporter; FABP: Fatty acid-binding proteins

Depending upon their requirements in the diet, nutrients are classified into macro- and micro-nutrients. The former are required in large amounts, while the latter are needed in traces. The absorption of nutrients occurs in the gastrointestinal (GI) tract. The transport of nutrients from the intestine is vital for the normal functioning of the living system. The ability to absorb nutrients in the small intestine depends on the surface area of the mucosa, the epithelium’s passive permeability, and the functional properties of intestinal nutrient carriers [17]. Digestion and assimilation of dietary ingredients take place through GI enzymes and transporter proteins articulated in the apical membrane of intestinal enterocytes. It has already been established that heat stress reduces feed intake in chickens [2, 10]. Acute heat stress tends to decrease the weight and length of the jejunum [8]. This indicates that heat stress directly affects the intestinal morphology to modify nutrient uptake (Fig. 2). Although high ambient temperatures modulates the expression of the genes responsible for transport of macronutrients, little information is available in terms of micronutrients. To understand the potential mechanisms of transport of nutrients and intestinal health under heat stress, several genes related to nutrient transport have been discussed below.

### Carbohydrates

To sustain constant energy supply, the uptake of carbohydrates from the intestinal lumen is important and is influenced by luminal and apical membrane digestion [18]. Glucose absorption during heat stress is crucial, as it is the main source of energy in most feeds. It is primarily transported in epithelial and nonepithelial cells by a sodium-dependent glucose transporter (SGLT) and glucose transporter (GLUT) systems, respectively. SGLT1 is responsible for Na^+^ −dependent glucose and galactose uptake across the brush border membrane and is considered to be the primary mediator of glucose assimilation in the small intestine [19]. The uptake of glucose decreases under heat stress conditions and remains constant for several days. This is evident from previous studies where the expression of the SGLT1 gene in *P. major* and ileum tissue decreased after 1 day of exposure when broilers were heat-stressed at 35 °C for 19 days [9]. Furthermore, the expression of the intestinal SGLT1 gene remained lower until the 7^th^-day after birds were exposed to heat stress at 35 °C for 7 days [5]. The effect of heat stress minimizes with time and can be justified with no variation in the expression of the SGLT1 gene in *P. major* and ileum tissue after 12 days of heat stress at 35 °C for 19 days [9]. The possibility of modulation of SGLT1 could also be related to the decrease in feed intake. To elucidate the relationship between SGLT1, feed intake, and heat stress, responses under pair-fed conditions would be of great importance. Expression of jejunal SGLT1 was significantly increased in chickens fed ad libitum under heat stress at 30 °C for 14 days; chickens were fed either similar amounts (pair-fed) of feed consumed by heat-stressed chickens or ad libitum feed kept at normal temperature (20 °C) for the same period [8]. Enhanced SGLT1 expression in the heat-stressed chicken compared to its counterparts suggested temperature-specific effects on modulating the uptake of nutrient transporters, irrespective of reduced feed consumption. The response could be a temporary adaptation of the body to meet the enhanced energy requirement under high-temperature conditions.

The commonly explored sodium-independent glucose transporter (GLUT) systems are GLUT2 and GLUT5. GLUT2 is responsible for transporting carbohydrates such as glucose, galactose, and fructose across the basolateral membrane, whereas GLUT5 controls the transportation of fructose across the brush border membrane [20]. Heat stress alters intestinal health depending on both temperature and duration for which the birds are exposed. Physiological effects such as the reduced surface area of the small intestine arising from decreased feed intake under heat stress may modify carbohydrate uptake through glucose transporters. This is evident from studies where heat stress reduces the expression of glucose transporter genes in the *P. major* and intestinal tissues when broilers were reared at 35 °C for 12 days [9]. In a recent study, the expression of the jejunal GLUT2 gene decreased in broilers in response to continuous chronic heat-stress at 35 °C for 7 days [5]. This suggests that the uptake of glucose and galactose is adversely affected when heat stress persists for several days.

### Amino acids

Excitatory amino acid transporters (EAATs) belong to the family of glutamate transporters. The EAAT3 gene is a sodium-dependent transporter that has a high affinity for anionic amino acids such as aspartate and glutamate [21]. It plays a significant role in providing vitality to intestinal cells, fortifying cellular proliferation, protein expression localization (crypts and lower villi), and diminishing profusion toward the villus tip [22, 23]. The jejunal expression of the EAAT3 gene was increased when chicks were exposed to 35–39 °C for 1–5 days [24]. It has already been established that excitatory amino acids such as aspartate and glutamate act as the primary fuel for enterocytes [25]. The enhanced expression of EAAT3 gene-mediated aspartate and glutamate uptake may help in maintaining intestinal permeability and enterocyte number that are adversely affected by heat generated tissue damage. The expression of aspartate and glutamate-related genes is inversely related to growth performance and could be the reason for the decrease in body weight of heat-stressed chickens [2, 26].

Peptide transporter 1 (PepT1) is a H^+^-dependent dipeptide and tripeptide transporter expressed in the brush border membrane of enterocytes [27]. Although other amino acid transporters exist, the transportation of most amino acids takes place through PepT1, due to increased efficiency than through the free amino acid transporters [28]. PepT1 expression in the intestine depends on dietary changes, the quality of feeds, and the stage of bird development [29]. The absorption of amino acids takes place in the intestine and decreases under heat stress conditions. This can be explained by the reduction in the expression of PepT1 in the ileum of the broiler chickens after 1 day of heat stress when birds were exposed to 35 °C for 19 days [9]. Consequently, the quantity of oligopeptides present in the ileum also decreased. This reduction is mediated by decreased protein utilization under deprived feed conditions in heat-stressed chickens [2]. In contrast, the jejunal expression of PepT1 was not affected when birds were exposed to 32 °C for 7 days [11]. The reason underlying this observation remains unclear. However, to achieve homeostasis, other mechanisms may be activated to sustain the amino acid levels.

### Fatty acids

Two types of fatty acid-binding proteins (FABPs) are found in the intestinal epithelium (I-FABP/FABP-2) and liver (L-FABP/FABP-1) [30]. FABP functions to participate in the absorption of long-chain fatty acids from digesta in the small intestine into intestinal epithelial cells and transport fatty acids from the cells to the organism for triglyceride synthesis [31]. Lipid peroxidation is the oxidative degradation of lipids and increases under stress. The expression of FABP is increased under cold stress to inactivate lipid peroxidation of the cell membrane and promote fatty acid storage in cold environment [32]. However, heat stress negatively impacts the expression of FABP. Several studies have reported a decrease in the intestinal expression of the FABP gene in chicken, independent of exposure time and intensity of stress [5, 9, 11]. It has already been established that nutrient absorption depends on the mucosal surface area and epithelium permeability in the intestine [17]. Furthermore, heat stress has been associated with modified intestinal barrier integrity and hyper-vascular permeability-mediated tissue damage [10, 33]. In conclusion, the reduction in the expression of the FABP gene under heat stress could be due to structural damage and epithelial loss of the intestine [8]. Moreover, the uptake of lipids by the enterocytes for re-esterification and transportation in the lymphatic system is negatively affected by structural damage. As a result, reduction of long-chain fatty acid absorption ultimately leads to a decrease in plasma triglyceride concentration in chickens [34].

The outcomes of the above studies suggest that the expression of SGLT1, PEPT1, and FABP genes decreases due to the reduction in feed intake and hypervascular permeability mediated intestinal damage in heat-stressed chickens. The expression of the EAAT3 gene increases to maintain gut permeability. However, the relationship between decreased feed intake and expression of the aforementioned genes during pair-fed conditions requires further exploration.

Vitamin and minerals play important roles during heat stress. Supplementation of dietary vitamins and minerals has positive effects in combating heat stress in poultry [1, 35, 36]. They mitigate the harmful effects of heat stress in various ways, such as modifying antioxidant enzyme levels and heat shock proteins. Thus, they are also recommended to be added to poultry diets when birds are reared in a tropical environment. Although numerous genes have been evaluated for the transport of these nutrients in mammals, including humans [37], to the best of our knowledge, a dearth of evidence exists regarding the role of these genes under heat stress. The possibility of heat stress to modulate micronutrient transporters in the GI tract cannot be ruled out as their expression is modulated by age, intestine, and feeding condition [38].

## Defense systems

To protect the body from the adverse effects of heat stress, a defense mechanism is activated in chickens. This happens in a series of step by step responses. Initially, the early response system stimulates the central nervous system. The message from the brain is transmitted to different parts of the body through neurons. Furthermore, the antioxidant enzyme system and the immune system come into play. Thus, regulation of relevant genes under heat stress can act as a marker to identify the severity of stress.

### Early response systems

**Fig. 3:**
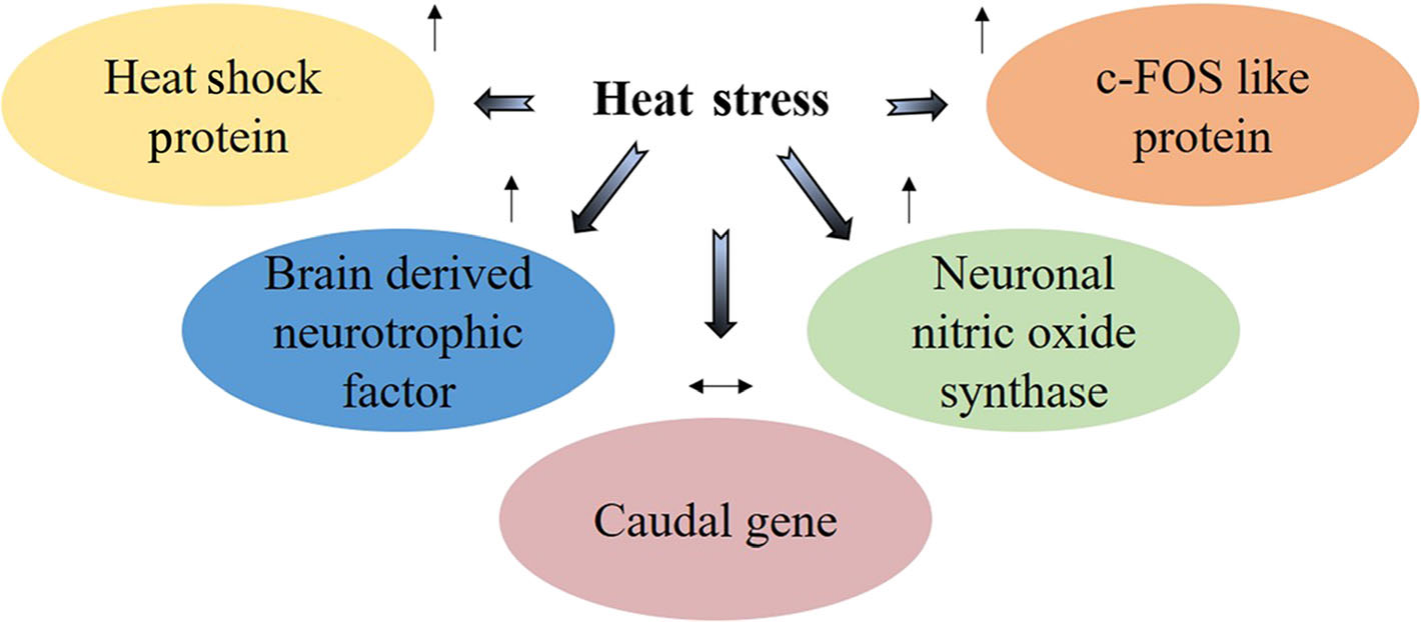
Consequences of heat stress on the chicken early response system. The gene expression of heat shock protein, brain-derived neurotrophic factor, c-FOS like protein and neuronal nitric oxide synthase is increased (↑) while the expression of the caudal gene is yet to be established (↔). The diagram is drawn based on the articles [40–43]

HSP70 and HSP90 are the members of most conserved and well-studied HSP family, and their expression is influenced by a variety of agents or stressors [39]. Heat is the foremost inducer of HSP-related genes (Fig. 3). Thus, expression of HSP70 and HSP90 has been studied extensively and used as a marker for heat stress in chickens [35, 39]. The HSP70 gene is known to play a role in protecting the body from the deleterious effects of oxidative stress [44], whereas HSP90 interacts with client proteins during the later stages of folding and modifies their configuration [45]. The expression of the aforementioned HSP-related genes and antioxidant enzymes is associated with heat preconditioning [46]. The ease of estimating the expression of HSP70 in different tissues such as the liver, heart, breast, brain, and lungs of broiler chickens also merits their use to evaluate heat stress. Each tissue responds differently to temperature modulation [47]. Leandro and his team reported 2 to 5 times higher HSP70 expression in the brain compared to other tissues [48]. The reason behind this could be related to the rapid accumulation of HSP70 in the brain under temperature-mediated stress [47]. Studies conducted on chickens revealed that the expression of HSP70 is also increased in the liver and monolayer of blood cells under high ambient temperature conditions [49, 50]. A possible explanation for their enhanced expression is that under high temperature conditions, the expression of HSP-related genes is influenced by heat shock factors, which activate the upstream promoter sequences for genes encoding heat shock protein [51, 52]. During hyperthermia, an individual’s body temperature is elevated beyond normal range due to failed thermoregulation, which happens as a result of overheating. Expression of HSP70 in peripheral leukocytes is induced significantly in broiler chickens when they are exposed to hyperthermal conditions of 45.4 °C [40]. In this condition, cellular defense mechanisms are activated through nuclear factor (NF)-κB mediated by pro-inflammatory cytokine induction [53].

Supplementation of nutrients, including vitamins and minerals is effective in controlling the expression of HSP70. Various antioxidants have been used to ameliorate the effects of heat stress on animals. The consequences of heat stress were minimized by supplementing selenium in turkey embryos. Selenium supplementation helped in sustaining the hepatic HSP70 expression which was otherwise enhanced when birds were kept at 40 °C for 2 h [35]. Organic selenium (selenium yeast) fed broilers challenged with enteropathogenic *Escherichia coli* also resulted in lower hepatic HSP70 under heat stress at 40 °C for 1 h, demonstrating the capability of selenium to condense heat stress responses even under challenging conditions [36]. Furthermore, dietary vitamin C supplementation reduces the expression of HSP70 in heat-stressed chicks. The role of antioxidants in altering the expression of proinflammatory and antioxidant genes has already been established [54]. The decrease in heat stress in selenium — and vitamin C— supplemented chicks could be attributed to pro-inflammatory cytokines and antioxidant modifications.

The brain is the controlling organ of the avian body that regulates temperature through neural circuits in the anterior hypothalamic region, and connections among circuits still need to be defined [55]. Its function is related to observing the local temperature changes and integrating temperature information from the periphery. During temperature control, there is a nonstop adjustment within the scopes between cold- and warm-sensitive cells [56]. Three types of neurotrophic variables are known in chicks: nerve growth factor, brain-derived neurotrophic factor (BDNF), and neurotrophin-3 among which BDNF is crucial for evolving plasticity [57]. BDNF is a neurotrophin and its level in a cell changes under unpleasant conditions related to psychosocial and physical factors (Fig. 3). It plays an active role in the survival of neurons by invigorating the differentiation and advancement of new neurons [58]. In chickens, BDNF behaves differently during various temperature manipulations. Its expression was expanded seven-fold and three-fold respectively, when 3-day-old chicks were exposed to high (37.5 °C) and low (30 °C) temperatures for 6 h indicating its role in induced adaptation [41]. It is also known to facilitate reconditioning under heat exposure when chicks were previously conditioned. Adaptation to reconditioning might be related to memory consolidation resulting from the initial acquisition of previous thermal exposure [57].

c-FOS is a nuclear phosphoprotein encoded by the proto-oncogenic c-fos and is one of the immediate-early genes. The c-FOS protein dimerizes with the Jun protein to form the transcription factor AP-1 that binds to different DNA sites and thus influences the transcription rate of the late effector genes [42]. It is well known that the hypothalamic-pituitary-adrenal (HPA) axis is the major brain circuit involved in stress responses in numerous distinctive species, including chicken. The action of the HPA axis is intervened through the paraventricular nucleus under several stress conditions together with temperature modifications. This can be justified from previous studies conducted on male broilers revealing enhanced immunoreactivity of c-FOS under the execution of social stressors [59]. The difference in the hypothalamic c-FOS activity patterns was also reported after a shock probe stressor test in rats [60]. The expression of hypothalamic c-FOS under stress demonstrated the actuation of several neurons at one time that appears to gradually develop during pre- and post-hatch ontogeny. This can be explained by the higher hypothalamic expression of the c-FOS gene on the day of hatch acting in a temperature-dependent manner when eggs were incubated at 35, 37.5, or 38.5 °C during the last week of incubation [56]. Furthermore, alterations in neuronal hypothalamic thermotolerance have been reported in warm or cold exposed Muscovy ducks during the last days of incubation studied in neurons through a glass electrode and spike 2 computer program (Cambridge Electronic Design Limited) [61]. In the final days of embryonic development, increment in incubation temperature initiated a long-lasting warm adaptation against post-hatch acute heat exposure at 42 °C for 1.5 h by significantly modifying the neuronal c-FOS expression in birds [42]. Thus, c-FOS is expected to be a sensitive marker for lifelong temperature adaptation in the central nervous system.

Neuronal nitric oxide synthase (nNOS) is pivotal for coordinating various physiological capacities for the improvement of organisms. It plays an imperative role in thermoregulation by tempering the feeding pattern, water, and energy balance [62, 63]. Most of its capacities have interceded through the hypothalamic region [64]. The action of nNOS is more articulated at lower temperatures and is clear from the former studies where normal and warm stimulated birds showed immature nNOS neurons and increment in nNOS activity improved compliance with the cold environment in ducks [43]. Furthermore, it was also suggested that durable cold stimulation compared to warm stimulation essentially persuades the nNOS action due to cold tolerance of the embryo [65]. Another study demonstrated enhanced expression of nNOS in jejunum nerve fibers of sodium nitroprusside (nitric oxide donor)-treated chicks [66]. From the above reports, it may be predicted that nNOS stimulation through either temperature stimulation or nitric oxide donor supplementation could be beneficial to encounter the effects of heat stress. However, little information is available on the relationship of nNOS in terms of embryonic or post-hatch supplementation to encounter temperature stress in chickens.

The caudal gene (Cdx) is a homeobox gene that encodes nuclear transcription factors involved in remodeling and cell differentiation [18]. The Cdx-A (previously CHox-cad) gene of the Cad family was first reported in chickens and found to be activated at a very early stage of incubation, gastrulation, gut closure, intestinal epithelial morphogenesis, enterocyte development, maturation, and post-hatch maintenance [67]. It is believed that it can play a key role in identifying the gut health and helping to replace aged cells. Further, it is suggested that the jejunal expression of Cdx-A is relatively lower on the 15th day of incubation and increases through developmental stages until after hatching [67, 68]. Thus, it can be predicted that its expression levels can be used to study intestinal stress in chickens. The adverse effects of environmental stress on growth performance and gut health due to a decrease in feed intake is already well documented. The intestine is the first digestive system to be directly affected by changes in nutrient intake and also shows the fastest and most dramatic changes in response to nutrient deprivation [69]. In addition, starvation for 48 h after hatching resulted in decreased intestinal CdxA gene expression, which was reversed after supplementation with feed [67]. This indicates that CdxA expression can prove to be an effective marker to study the adverse effects of environmental stress in chickens. Furthermore, due to the morphological complications and challenges arising through the closed environment of the egg, its importance will be comparatively higher during different stages of embryogenesis and can prove to be effective for studying either thermal manipulation or *in ovo* supplementation.

Taken together, these studies suggest that the expression of HSP-related genes is expressed in various tissues with a tendency to highly upregulate in the brain. The hypothalamic gene expression profiling of the BDNF and c-FOS genes could effectively be used to evaluate the possibility of adaptation to the adverse environment. The intestinal expression of the CdxA gene facilitates the identification of chick sensitivity against food irrespective of nutrient uptake at a very early age during temperature manipulation. Thus, these genes alone or in combination can be used as effective markers to evaluate the stress level with higher possibilities to even acknowledge the recovery under treatment conditions.

### Antioxidant enzyme systems

**Fig. 4:**
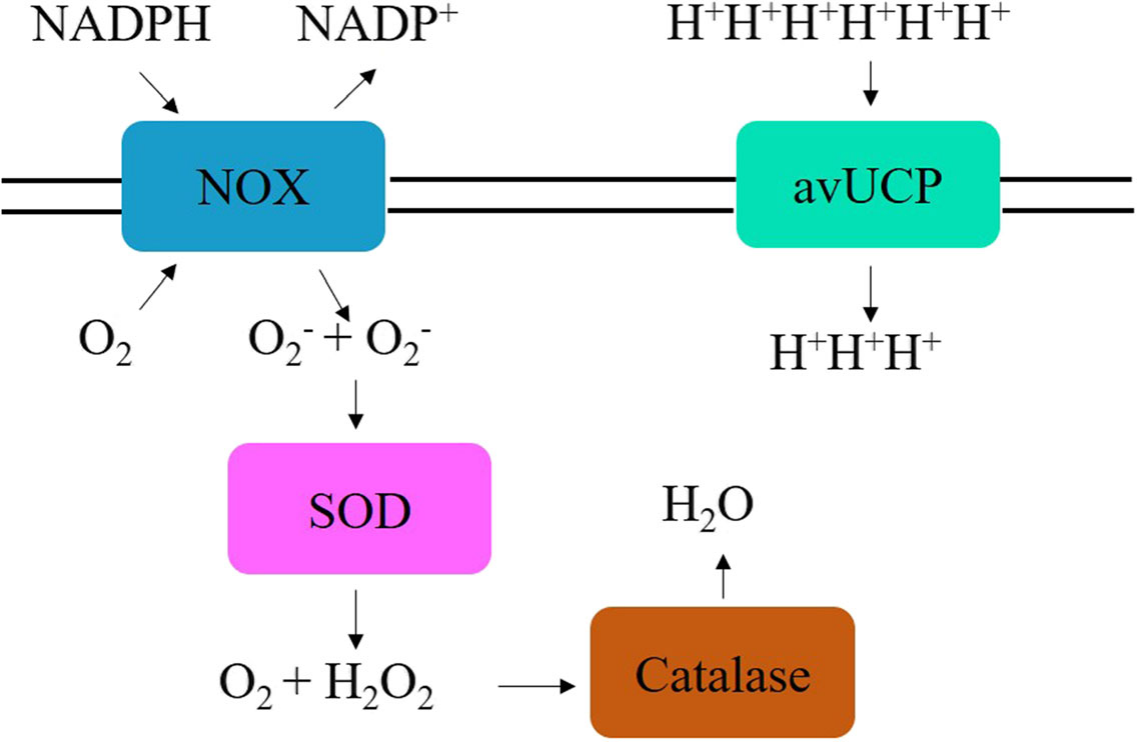
Mechanism of antioxidant enzyme systems in response to heat stress in the chicken. Production of reactive oxygen species through NOX is catalyzed to hydrogen peroxide by SOD and further broken down to the water through CAT in the presence of hydrogen ion maintained by avUCP. With the modification of the diagram [71]. NOX: nicotinamide adenine dinucleotide phosphate oxidase; SOD: Superoxide dismutase; CAT: Catalase; avUCP: Avian uncoupling protein

Oxidative stress is characterized by the production of excessive ROS [70]. High ambient temperatures during the rearing of broiler chickens induce oxidative injury [3]. Several effective antioxidant systems work side-by-side to prevent oxidative damage (Fig. 4). Among them, CAT, SOD, and GPX are the major antioxidant enzymes [72]. SOD catalyzes the dismutation of superoxide radicals to hydrogen peroxide, while CAT catalyzes the breakdown of hydrogen peroxide to water [73]. Previous studies have demonstrated that exposure to high ambient temperatures causes a compensatory increase in the activity of SOD, GSH-Px, and CAT in the serum, liver, and muscle of broiler chickens [4]. The mechanism behind the increase in these enzyme systems is critical.

Exposure to increased temperature generates excessive free radicals, and to counter its forfeits, short-term protective action is implemented through the antioxidant system with the release of enzymes such as SOD, GSH-Px, and CAT [74]. Recently, it has been reported that hepatic expression of the SOD gene is upregulated in thermally stressed broilers at 36 °C for 6 h [15]. SOD is the foremost basic defensive enzyme that helps in preserving highly reactive O_2_^−^ to the required levels in the body by eradicating superoxide anion free radicals by converting high reactive O_2_^−^ to low reactive H_2_O_2_ [75]. Enhanced SOD expression during the initial stage of exposure suggests its protective role through hydrogen peroxide-mediated O_2_^− ^reduction. Products from plant origin are known to possess secondary metabolic substances with strong antioxidant ability [76]. Supplementation of essential oils from plant origin enhanced the expression of the SOD gene in thermally exposed broilers [77]. This can be explained by the enhanced antioxidant capacity in essential oil-treated chicks that modulate SOD. Similarly, hepatic expression of the CAT gene is also upregulated under heat stress [16]. CAT is a part of the antioxidant system, along with SOD, and functions in stabilizing the production of excessive ROS generated in response to heat stress. It may facilitate the breakdown of hydrogen peroxide produced by SOD to water and molecular oxygen (Fig. 4). In contrast, long-term heat exposure results in decreased expression of antioxidant enzymes due to the formation of tissue injury, lesions in cells, and secretion of antioxidant cofactors such as Zn, Cu, Se, vitamin C, and vitamin E [78].

NADPH oxidase 4 (NOX4) is a membrane-bound complex that protects tissues from inflammatory stress by acting as an oxygen sensor and comprises 7 isozymes [79]. Nevertheless, it is essential to maintain ROS levels in the cells; otherwise, excessive ROS can cause oxidative damage. Previous studies suggested that NOX4 is the source of ROS during periods of heat stress and superoxide (O_2_^−^) levels are positively correlated with NOX4 expression *in vitro* [13, 79]. They also reported enhanced expression of NOX4 mRNA under heat stress. The determination of ROS generation through the NOX4 gene can be beneficial for evaluating the effects of heat stress in chickens. The enhanced expression of the NOX4 gene in avian muscle cells cultured at 41 °C followed by a decrease in its expression after supplementing superoxide scavenger (4-hydroxy-TEMPO) indicates that it can be used as an effective marker in heat stress studies [13]. The possible action of NOX4 could be through transferring an electron from NADPH after coming in contact with the oxygen to produce NADP^+^ and O_2_^−^.

avUCP gene codes for uncoupling proteins and are usually present in the inner mitochondrial membranes, which play important roles in proton regulation. It is believed that avUCP could play a key role in thermogenesis by controlling the production of ROS and protecting the cell against its deleterious effects [80]. The role of avUCP is mediated through proton regulation [71]. Indeed, a protective role of avUCP was found against ROS production in cold-stressed ducklings [81]. Furthermore, an increase in the ROS level is associated with the down-regulation of muscular avUCP in heat-stressed chicks [14]. Since the mitochondrial membrane is composed of phospholipids containing fatty acids, a direct relationship exists between fatty acids and uncoupling proteins. It has already been reported that heat is the source of ROS production and the expression of the FABP gene decreases under heat stress [4, 9, 11]. Thus, the correlation between the expression of avUCP and FABP is established and can be confirmed by the decrease in the expression of these genes under heat stress conditions.

Contemporaneously, it can be suggested that antioxidant enzymes help lower the adverse effects of heat stress, and their activities can be observed through enhanced gene expression of SOD and CAT in chicken. However, this response is short-lived and loses its characteristics against chronic heat stress due to the formation of tissue injury. Furthermore, the expression of NOX4 is an effective marker for predicting heat stress through ROS generation in chicken.

### Immune system

Immunity is an important aspect of poultry because of its direct relationship with production performance. Previous reports have suggested that immunity is suppressed under high ambient temperature [82, 83]. Cytokines and Toll-like receptors (TLRs) are important markers of immunity. Cytokines imply immune regulation through hematopoietic cells and facilitate host defense and homeostasis [84]. They primarily comprise interferons (IFNs), interleukins (ILs), transforming growth factors (TGFs), tumor necrosis factors (TNFs), and small peptide chemokines. Similarly, TLRs have distinctive roles, including recognizing pathogen-associated molecular patterns (PAMPs) and antigen presentation [85]. Heat stress-mediated suppression of the chicken immune system takes place through gene regulation. This could be the result of many different underlying mechanisms. Alteration in the intestinal barrier integrity resulting in modified microbiota under heat stress could be one of the reasons [10]. This may allow the penetration of pathogens through the intestinal epithelial membrane, resulting in enhanced antigen presentation through TLRs to the native T cells. Furthermore, antigen-specific immune responses and inflammatory responses generated through enhanced cytokines and interleukins may result in hypervascular permeability-mediated tissue damage [33].

**Fig. 5:**
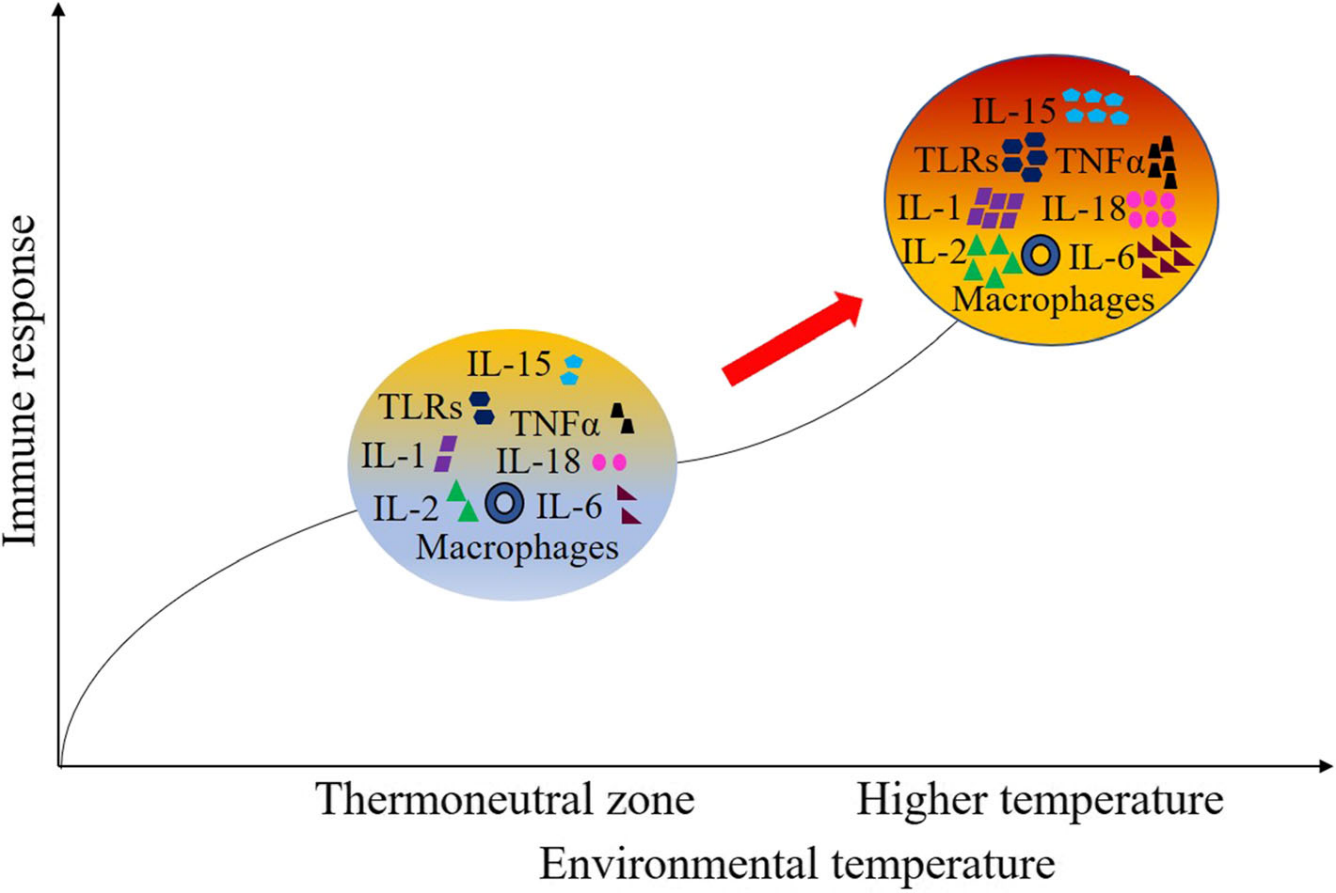
Effects of aberrant temperature during chicken rearing result in immunomodulation. Heat stress enhances the expression of various proinflammatory cytokines, toll-like receptors, or other interleukins either alone or mediated through macrophages in chickens. The diagram is drawn based on the articles [6, 7, 12]

Cytokines are essential immune gene coding proteins that have been recognized as endogenous signaling molecules interfering with the cellular defense framework against the inflammatory response actuated by increased temperature [86]. ILs are among the group of cytokines (naturally occurring proteins) that have a major role in stimulating immune responses and inflammation (Fig. 5). They have been classified from IL-1 to IL-17, with a specific role for each type. Furthermore, IL-1, IL-2 IL-6, IL-18, and TNFα belong to the pro-inflammatory cytokine family. They have been known to play an active role in the inflammatory response under high ambient temperature [87]. The expression of these pro-inflammatory cytokines are enhanced under heat stress, presumably via activating immune functions through an enhanced proliferation of lymphocytes and macrophages under high ambient temperature [1, 6, 12]. Excessive expression and enhanced proliferation may further result in tissue damage. It is well known that vitamins help to alleviate the negative effects on growth performance and immunity in response to heat stress due to their suppressive action against the expression of pro-inflammatory cytokines [1, 88]. Thus, in the tropical region, it is highly recommended to supplement these antioxidant vitamins in the diet. The role of IL-15 in the growth and proliferation of T-cells, B-cells, intestinal epithelium, and natural killer cells has already been established [89]. In a recent study, it was reported that acute heat stress at 40 °C for 7 h tends to enhance splenic expression of the IL-15 gene in chicken [7]. This indicates that IL-15 quickly reacts to heat stress to help the chick maintain homeostasis by proliferating immune cells.

The genes for TLRs are a highly conserved group of DNA molecules in the living system [90]. Pathogens contain small conserved molecular motifs known as PAMPs. TLRs play a crucial role in protecting the innate immune system through the recognition of PAMPs [85]. TLRs are activated by numerous components, such as antiviral compounds and single-stranded RNAs, and are implicated in the immune response to viruses such as influenza that can be prominent during stress conditions [90]. It has already been established that the GI tract responds to heat stress in a variety of ways, including alterations in the microbiota and intestinal barrier integrity [10]. The expression of TLR-4 gene was increased in the spleen and intestinal (jejunum and ileum) tissue of broilers reared at 38–39 °C for 6 h per day until 5 days of heat stress [6, 12]. This may be due to the impairment of intestinal health thus allowing luminal pathogens to penetrate through the intestinal epithelium and generate an innate immune response. As a result, TLR signaling is activated, resulting in inflammation [91]. In addition to the splenic tissue, the presence of the TLR-4 gene was also reported in different locations of the intestine. The expression of TLR-4 gene was higher in the ileum than in the jejunum under heat challenge [12]. Thus, it can be proposed that heat-induced damage is more severe in the ileum than in the jejunum. The severity could be related to the microbiota composition, which differs in both the location (ileum and jejunum) of the intestine [92]. Along this, it can also be suggested that the modification in the expression of TLR-4 gene relates to the alteration in the permeability of the intestine arising from heat stress.

Transforming growth factor-beta (TGF-β) is multifunctional homo-dimeric protein belonging to the TGF superfamily. It is secreted by various cell types in mesenchymal, epithelial, and neuronal tissues. TGF-β present in thymocytes and thymic stromal cells may regulate the ability of immature thymocytes to progress through the cell cycle and differentiate into CD3+ [93]. The expression of TGF-β decreased in the cecal tonsil when broilers were reared at 31 ± 1 °C for 7 days (35 to 41 days) under heat stress [83], whereas it increased in heat-stressed chicks supplemented with copper oxide nanoparticles [82]. TGF-β can upregulate the expression of Hsp90 and Hsp70 in cultured chicken embryo cells [94]. This indicates that the role of TGF-β in modulating immunity is regulated through HSP. HSP is subsequently known to activate the expression of several other pro-inflammatory cytokines [88]. Thus, the possibility of correlation of TGF-β with other pro-inflammatory cytokines to modulate heat stress also exists. However, the role of heat stress in modulating TGF-β still needs to be established.

Based on the above facts, it can be predicted that the expression of ILs and TLRs increases under heat stress conditions. The expression of splenic and intestinal TLR4 gene can be used as a marker under heat stress to predict pathogen penetration and immunomodulation in chickens.

## Conclusion

To improve livestock production, heat stress is one of the biggest challenges. The poultry industry is continuously working to reduce the effects of temperature variations. The evaluation of heat stress is mandatory for explaining its destructive impacts. Recent technological advances and the strategic implementation of molecular biology approaches enable a rapid understanding to encounter these challenges with higher efficiency. Although suppressed feed intake and body weight have been primary parameters for heat stress [1, 2], by evaluating the gene expression of macronutrient transporters, it is possible to have deep insights into the modulation of nutrient uptake in heat-stressed chickens [5, 9]. The roles of vitamins and minerals in alleviating heat stress remain to be assessed for a better understanding of this issue. The impact of heat stress in terms of exposure time (long or short term) and period (early, mid, and late phases of life) is a critical issue. The gene expression of nutrient transporters does not follow any sequential pattern during heat stress [5]. Nevertheless, enhanced upregulation of nutrient transporters, regardless of feeding conditions, implies that heat stress has more specific effects than previously anticipated.

Early responses to heat stress could be elucidated through specific genes. Among them, the expression of HSP-related genes is the most prominent and extensively studied. Antioxidant enzyme systems comprising SOD and CAT play a crucial role in the generation of ROS generated by heat stress. A series of actions take place to neutralize the NOX-produced ROS, such as SOD, catalase, and avUCP [71]. Immunity is also hampered under heat stress conditions and channeled through enhanced gene expression of cytokines and toll-like receptors [7, 8, 12]. Overall, heat stress has a drastic effect and can be evaluated through the aforementioned genes. These may further be selected as markers to evaluate the possession of heat stress in chickens. The present study has focused on evaluating the drastic effect of heat stress; however, recovery time is also an important aspect that can be evaluated through the above-mentioned genes and could be of interest for future research.

## Data Availability

Not applicable.

## Abbreviations

SGLT: Sodium-dependent glucose transporter
GLUT: Glucose transporter
EAAT: Excitatory amino acid transporter
PepT: Peptide transporter
FABP: Fatty acid-binding proteins
HSP: Heat shock protein
BDNF: Brain-derived neurotrophic factor
HPA: Hypothalamic-pituitary-adrenal
nNOS: Neuronal nitric oxide synthase
Cdx: Caudal gene
ROS: Reactive oxygen species
SOD: Superoxide dismutase
CAT: Catalase
GPX: Glutathione peroxidase
NOX4: Nicotinamide adenine dinucleotide phosphate oxidase 4
avUCP: Avian uncoupling protein
IL: Interleukin
TLR: Toll-like receptors
TGF-β: Transforming growth factor-beta
TNFα: Tumor necrosis factor-alpha
IFN: Interferon
PAMP: Pathogen-associated molecular pattern
